# Evaluation of pooling of samples for testing SARS-CoV- 2 for mass screening of COVID-19

**DOI:** 10.1186/s12879-021-06061-3

**Published:** 2021-04-17

**Authors:** Sally A. Mahmoud, Esra Ibrahim, Bhagyashree Thakre, Juliet G. Teddy, Preety Raheja, Subhashini Ganesan, Walid A. Zaher

**Affiliations:** 1 Biogenix G42 lab, Abu Dhabi, UAE; 2 G42 Health care, Abu Dhabi, UAE

**Keywords:** COVID-19, SARS-CoV-2, Diagnosis, Mass screening, Sample pooling

## Abstract

**Background:**

The current pandemic of the SARS-CoV-2 virus, widely known as COVID-19, has affected millions of people around the world. The World Health Organization (WHO) has recommended vigorous testing to differentiate SARS-CoV-2 from other respiratory infections to aid in guiding appropriate care and management. Situations like this have demanded robust testing strategies and pooled testing of samples for SARS-CoV-2 virus has provided the solution to mass screening of people for COVID-19. A pooled testing strategy can be very effective in testing when resources are limited, yet it comes with its own limitations. These benefits and limitations need critical consideration when it comes to testing highly infectious diseases like COVID-19.

**Methods:**

This study evaluated the pooled testing of nasopharyngeal swabs for SARS-COV-2 by comparing the sensitivity of individual sample testing with 4-and 8-pool sample testing. Median cycle threshold (Ct) values were compared, and the precision of pooled testing was assessed through an inter- and intra-assay of pooled samples. Coefficient of variance was calculated for inter- and intra-assay variability.

**Results:**

The sensitivity becomes considerably lower when the samples are pooled. There is a high percentage of false negative reports with larger sample pool size and when the patient viral load is low or weak positive samples. High variability was seen in the intra- and inter-assay, especially among weak positive samples and when more number of samples are pooled together.

**Conclusion:**

As COVID − 19 infection numbers and need for testing remain high, we must meticulously evaluate the testing strategy for each country depending on its testing capacity, infrastructure, economic strength, and need to determine the optimal balance on the cost-effective strategy of resource saving and risk/ cost of missing positive patients.

## Background

Since the outbreak in December 2019 in Wuhan, China, the world has been witnessing the most crippling pandemic in recent history. Widely known as COVID-19 and caused by the SARS- COV-2 virus, the new virus exhibits a variable incubation period, normally up to 14 days, and asymptomatic carriers can transmit the virus, allowing it to spread rapidly and affect larger numbers of people than either SARS or MERS, despite COVID-19 having lower case fatality rates [1]. All these characteristics of the novel virus have made the containment and control of the new virus challenging.

The World Health Organization (WHO) has recommended robust diagnostic testing to differentiate SARS-CoV-2 from other respiratory infections to aid in guiding appropriate care and management. Since the pandemic has affected millions of people, mass testing requires a lot of resources. Further the WHO has suggested around 10–30 tests per Positive COVID-19 case as a benchmark for adequate testing with a recommended positive rate lower than 10% [2].

In situations like this, sample pooling can be a vital strategy for testing in large numbers of people, in which sample extracts from a random number of samples from a heterogeneous population group are combined into a single tube for pooled polymerase chain reaction (PCR) analysis. This strategy has shown to be cost–effective during mass testing compared with individual testing, which is particularly important with limited testing resources [3].

When the disease prevalence is lower, it can be advantageous to pool individual samples into a single pool because this increases test capacity and reduces the number of PCR tests, and thus testing burden, required [4, 5]. For COVID-19, it has been estimated that using a pooling strategy reduces cost by 69% and requires tenfold fewer tests [6, 7]. Recent research has also established the optimal pool size that maintains testing accuracy for SARS-CoV-2 PCR is a pool size of up to 32 samples. This method has shown that costs can be reduced substantially without sacrificing accuracy [8–10].

However, when samples are diluted, there could be less viral genetic material available to be detected and this increases the likelihood of false negative results. On the other hand, studies have established that sample pooling greatly increases the number of individuals that can be tested while using fewer resources with only a small reduction in sensitivity; this may be acceptable depending on the pooling efficiency [11] Therefore, the FDA generally recommends that pooling test shows ≥85% percent positive agreement when compared with the same test performed on individual samples for the pooling method to be an acceptable method [11].

**Fig. 1:**
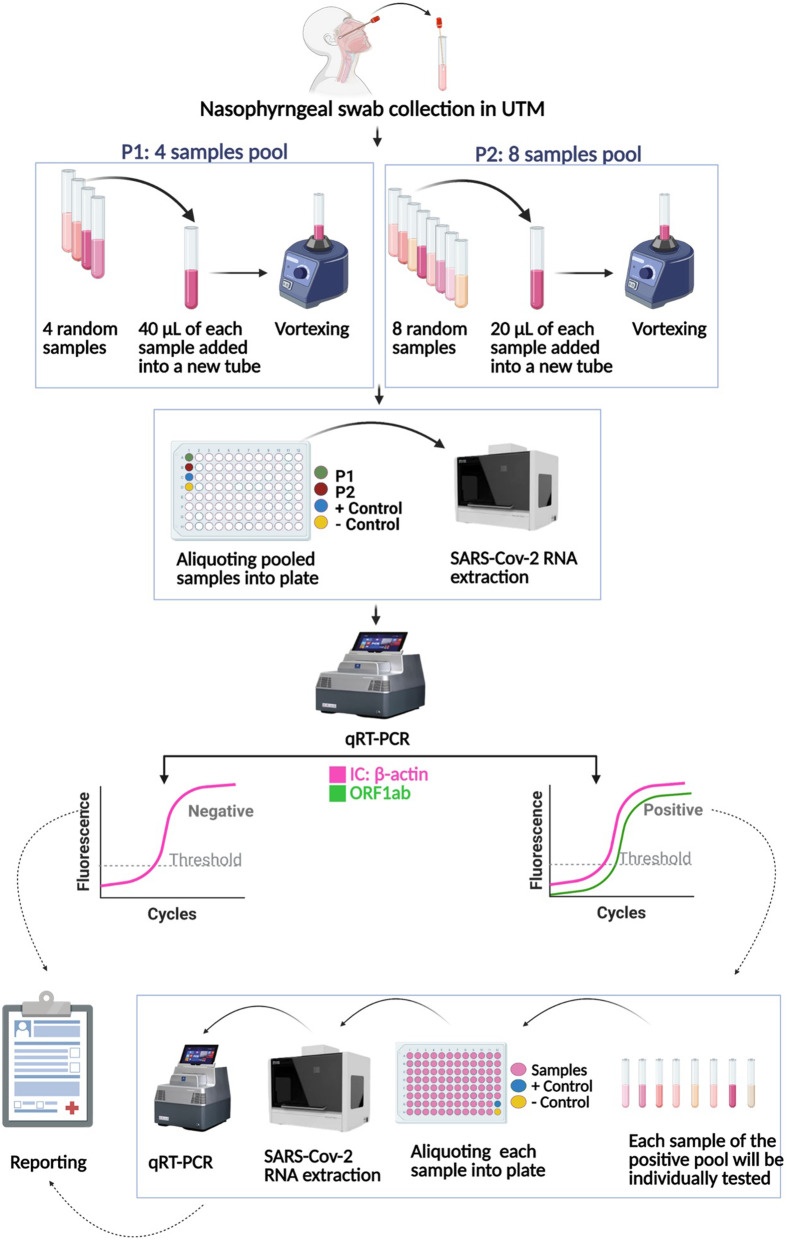
Sample pooling technique of SARS-CoV-2 detection

In the UAE, as of November 2020, there are around 1500 COVID-19 cases per 100 K people in the population and the UAE has the highest number of tests done per 100 K in the population when compared to other nation. Around 149,000 tests are done per 100 K population and the positive rate is 1% [12] (Fig. 1) This study aims to evaluate the pooling strategy for mass screening of COVID-19, which may be useful to further augment testing in the UAE and globally.

### Aim

The aim of the study is to evaluate the pooling method for mass screening of COVID-19.

### Objectives

- To evaluate the sensitivity of 4- and 8-sample pooling for testing COVID-19.
- To study the repeatability and reproducibility of sample pooling by doing an inter- and intra-assay precision analysis.

## Materials and method

The samples for this study were collected from individuals using nasopharyngeal swabs and were transported using viral transport medium (VTM). All methods were carried out in accordance with relevant guidelines and regulations. RNA extraction was performed on samples by the automated machine MGISP − 960. After the RNA extraction, 10 μl of the sample extract are added to 20 μl of the master mix (BGI RT-PCR fluorescence KIT). Every plate has a positive control and a negative or blank control.

For individual sample extraction, 160 μl of the sample were used in each well, while, for the pooled sample, the 160 μl of sample in each well was constituted by 40 μl of each sample in 4-fold pooled sampling and 20 μl of each sample for 8-fold pooled sampling. (Fig. 1).

Then the pooled samples are extracted following the Real-Time quantitative PCR (RT-qPCR) procedure. VIC channel represents the B-actin housekeeping gene as internal control while FAM channel represents the ORF1ab gene for SARS-COV-2 virus detection. A positive test specimen is one in which VIC has a cycle threshold (Ct) value ≤32 and FAM Ct ≤ 35, with both fluorescence curves S-shaped. A negative sample is one where there is no fluorescence curve at the FAM channel and a FAM Ct value of < 32 and S-shaped curve at the VIC channel.

If an S- shaped fluorescence curve is detected with a FAM Ct > 35, re-extraction is done and RT-qPCR test is repeated; if a negative result is detected, it is reported negative. If an S-shaped curve is detected with a FAM Ct ≤ 38 in re-extraction results, then the sample is reported positive.

### Sensitivity of pooled sample

For the sensitivity analysis, 40 known positive samples and 280 known negative samples were considered. In the 4-fold pooled sample testing, 40 known positive samples and 120 known negative samples were tested, while in the 8-fold pooled sample testing, 40 known positive samples and 280 known negative samples were tested. Each well contained one known positive and 3 known negative samples in the 4-fold pooled method and each well contained one known positive and 7 known negative samples in the 8-fold pooled sampling.

To analyse the sensitivity of samples with various viral load, out of the 40 samples used for pooling, 10 high viral load positive samples (HP) with Ct ≤ 20, 10 medium viral load positive samples (MP) with Ct > 20 to < 30, and 20 low viral load or weak positives samples (WP) with Ct > 30 were used.

The sensitivity of the 4-fold pooled sampling and 8-fold pooled sampling was determined by running the samples individually for both pooled methods and the ability to detect the true positives were compared.

### Precision

To assess the precision of pooled sample method intra- and inter-assay precision studies were conducted.

### Intra-assay precision

Intra-assay precision was calculated by running 30 known positive samples in 4 pool and 8 pool sampling, twice. Thirty known positive samples were classified into three groups of 10 samples, each based on the Ct values of HP, MP, and WP samples, as mentioned above.

All these samples were run twice, and the variability was assessed by calculating the coefficient of variation (CoV%).

### Inter-assay precision

Inter-assay precision was calculated by running 30 known positive samples, classified into three groups based on the Ct values as mentioned above (HP, MP, WP). These samples were run on three consecutive days and the variability was assessed by calculating the coefficient of variation (CoV %).

While doing the intra- and inter-assay experiments, three known negative samples were added to the same well along with one of the 30 known positive samples for 4-fold pooled samples and seven known negatives were added to the same pool along with one known positive for 8-fold pooled samples.

## Results

Our study found that the sensitivity of 4-pooled samples was 75% and the sensitivity of 8-pooled samples was decreased to 62.5%. In the low viral load, weak positive samples (Ct value ≥35), sensitivity of 4-pooled samples was 50% and it further reduces to 25% in 8-pooled sampling, with a false negative percentage as high as 75%. (Table 1).

**Table 1:** Sensitivity of pooling methods

Pooling methods	Standard method (individual samples)		Sensitivity (%) (95% CI)	False negative (%)
	Positive	Negative		
4-fold pooling				
Positive	30	0	75 (61.7–88.3)	25
Negative	10	0		
8-fold pooling				
Positive	25	0	62.5 (47.5–77.5)	37.5
Negative	15	0		
4-fold pooling of weak positive samples				
Positive	10	0	50 (28.09–71.91)	50
Negative	10	0		
8-fold pooling of weak positive samples				
Positive	5	0	2 (6.03–43.97)	75
Negative	15	0		

**Fig. 2:**
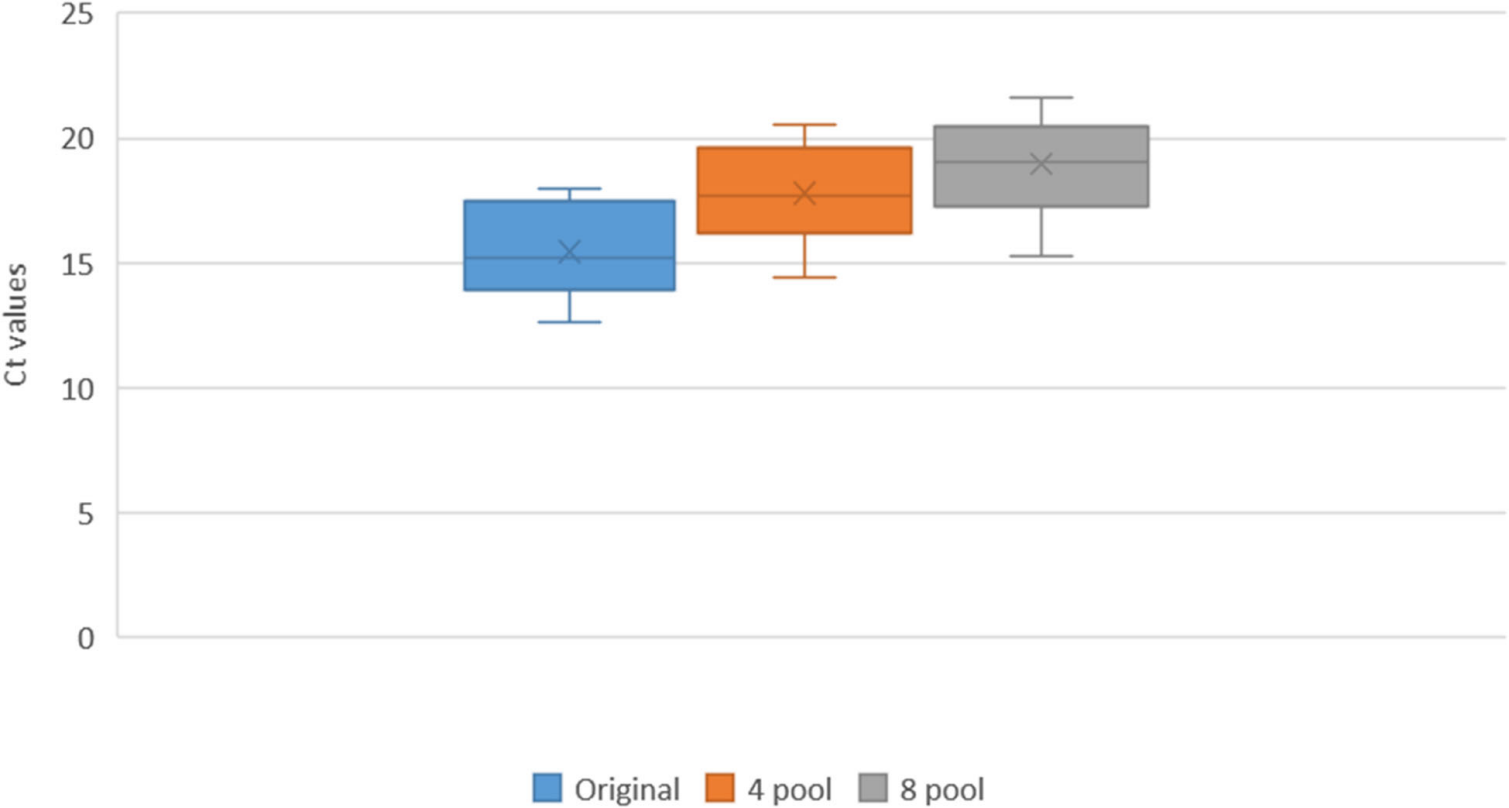
The Ct values of High viral load positive (HP) samples

**Fig. 3:**
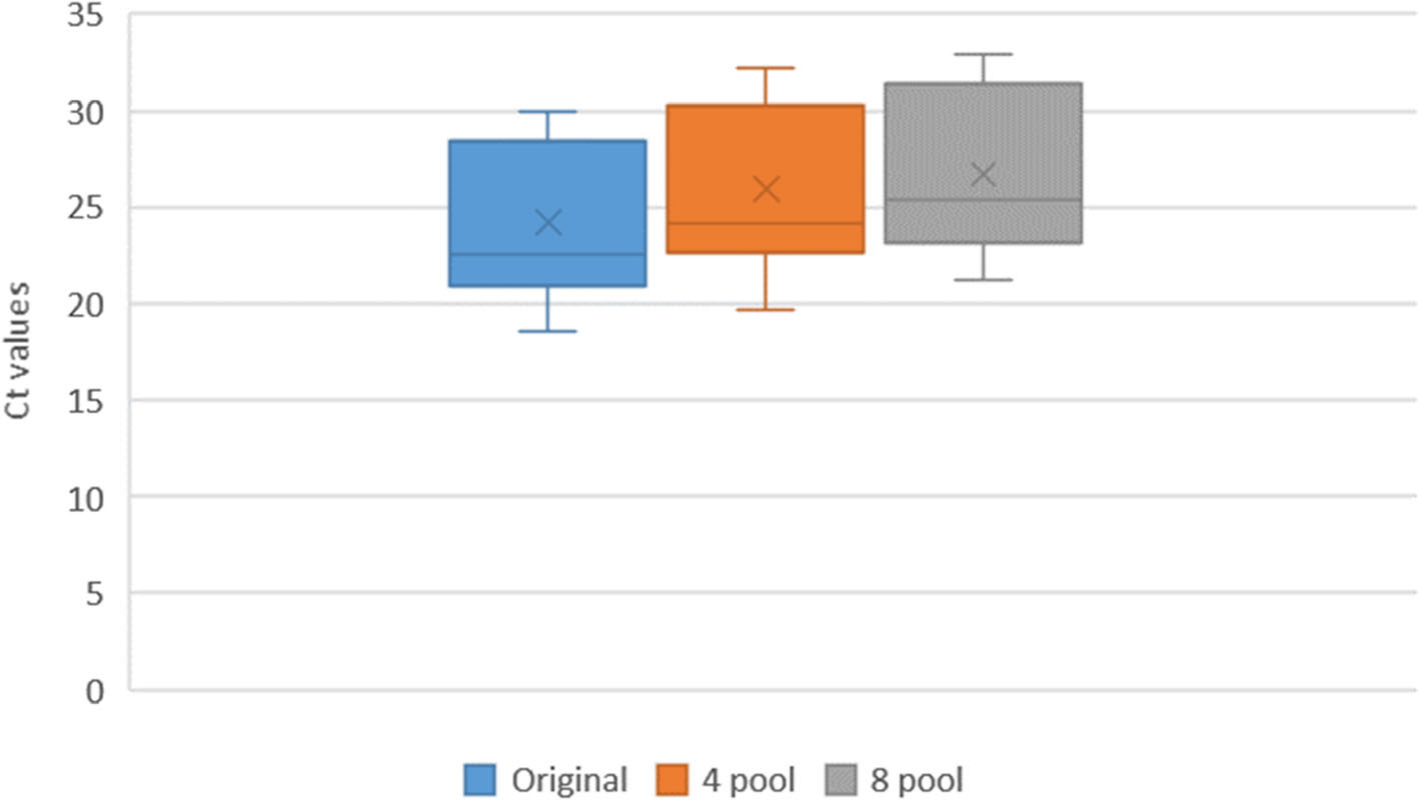
The Ct values of Medium viral load positive (MP) samples

**Fig. 4:**
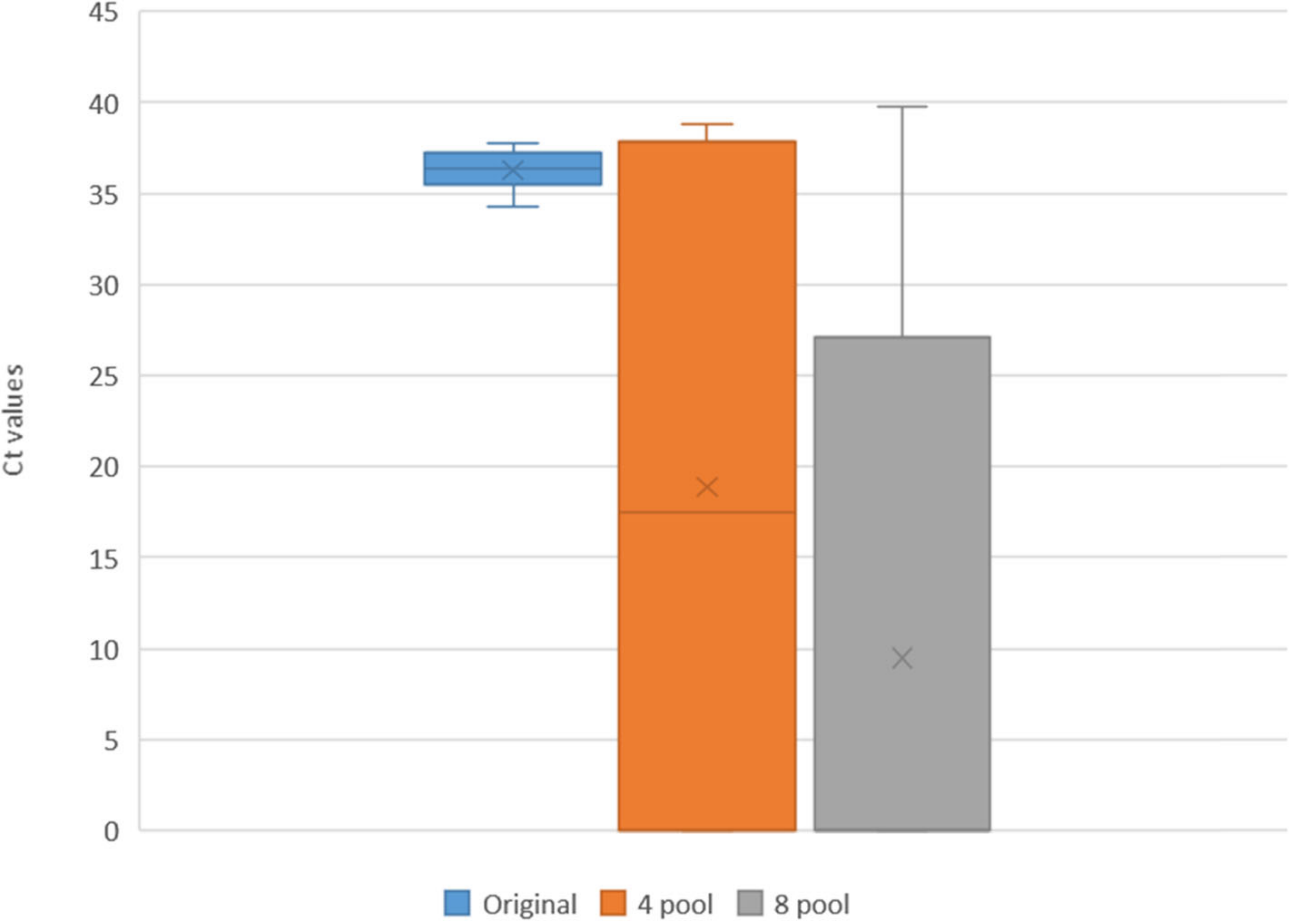
The Ct values of low viral load / weak positive (WP) samples

The Ct difference was calculated between individual and pooled samples and our study found the difference increased with the increase in pool size. This difference was more pronounced in the low viral load, weak positive pooled group. (Table 2). In the weak viral load positive sample pooling, the median Ct value became almost 0, as most samples turned negative with Ct value of 0 in the 8 pool of weak viral load positive sample. (Figs. 2, 3, 4).

**Table 2:** Median Ct difference between individual and pooled samples

Pool details	Ct difference (Median)	Interquartile range (IQR)
4 pool (HP)	2.29	0.49
4 pool (MP)	1.79	0.79
4 pool (WP)	- 18.26	39.60
8 pool (HP)	3.10	1.38
8 pool (MP)	2.66	1.21
8 pool (WP)	−36.01	28.81

* *HP* High viral load positive sample, *MP* Medium viral load positive sample, *WP* low viral load or weak positive sample

**Fig. 5:**
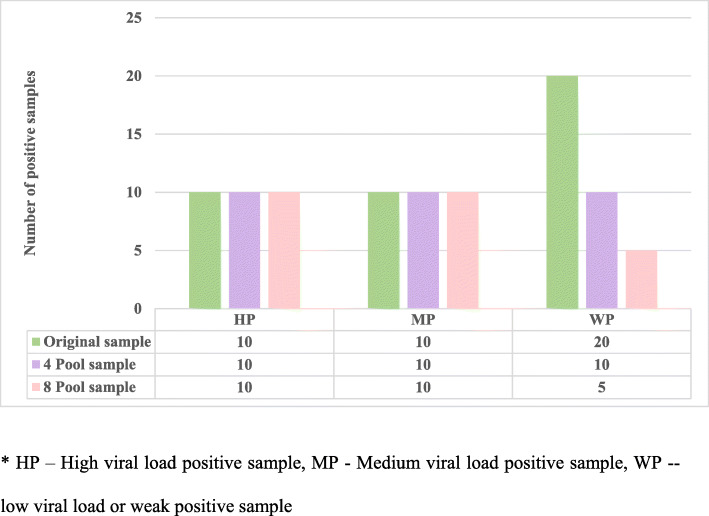
Result discrepancies of known positive samples in sample pooling testing strategy. * HP – High viral load positive sample, MP - Medium viral load positive sample, WP -- low viral load or weak positive sample

The discrepancies in results of the 40 positive samples (10 high viral load positive samples, 10 medium viral load positive samples and 20 low/weak viral load positive samples) were analysed and we found that the 10 high and 10 medium viral load samples were detected as positive when 4 and 8 sample pooling method were followed. However, in the low/weak viral load positive samples, 20 positive samples were tested, of which only 10 samples (50%) were detected positive in 4-pool sampling method. Only 5 out of these 20 samples were detected in the 8-pool sampling method hence missing 75% of the weak positive samples. There was a total of 15 positive samples that missed detection after the samples were pooled and all the missed positive samples belonged to the low/weak viral load positive samples. (Fig. 5).

The CoV % ranged from 0 to 4% in intra- and inter-assay of 4-pooled samples of high and medium viral load positive samples; however, inter -assay of 8-pooled samples and weak positive samples of 4-pool and 8-pool for both inter- and intra-assays were very high. (Tables 3 and 4).

**Table 3:** Intra- and inter-assay of 4 pooled sampling

4 – fold pooled sample	Coefficient of Variation %
Intra assay	
High viral load positive samples	0–2%
Medium viral load positive samples	0–2%
Low viral load/ Weak positives	2–141%
Inter assay	
High viral load positive samples	1–2%
Medium viral load positive samples	1–4%
Low viral load/ Weak positives	1–173%

**Table 4:** Intra- and inter-assay of 8-pooled sampling

8 – fold pooled sample	Coefficient of Variation %
Intra assay	
High viral load positive samples	0–4%
Medium viral load positive samples	0–3%
Low viral load/ Weak positives	2–141%
Inter assay	
High viral load positive samples	2–14%
Medium viral load positive samples	2–87%
Low viral load/ Weak positives	26–173%

## Discussion

Our study showed that the strategy of pooling of samples for COVID-19 RT-qPCR has lower sensitivity than the standard individual RT-qPCR and that the sensitivity decreases with the increase in the pool size. Further, when low viral load positive samples were pooled, the sensitivity was as low as 25%, which leads to increased false negatives reported. Similar studies have reported varied results; some studies have claimed pooling to be an effective strategy, including a study conducted in Malaysia that showed that pools of 10 samples were similar in sensitivity compared to individual testing [13]. A study in the UK has strongly supported pooled approach and reports a clinically insignificant sensitivity loss when samples are pooled and a false positive rate up to 5.3% [14]. But another study done in Spain evaluating the sensitivity of pooled samples testing various solutions and VTM showed that the sensitivity varied from 62.5 to 81% and false negatives were as high as 40% [15].

This study also showed that the pooling resulted in a median loss of 2.29 Ct for high viral load positive 4-pool samples to a median loss of 36.01 Ct in 8-pool low viral load positive samples. This is because the samples became negative with a Ct value of 0, after 8-sample pooling. This shows that in low viral load and weak positive pooled samples the cycle threshold increased or that most samples went undetected. A study in Kenya showed that the cycle threshold values were higher for samples that were pooled then tested individually [16].. A study in Spain also showed that sample dilution in pooling strategy resulted in a median loss of 2.8 to 3.3 Ct and thereby drop in sensitivity, which is similar to what was found in our study [17].

The coefficient of variation was high among the low viral load positive samples, both in the inter- and intra-assays. This shows a high variability in the results when the samples are pooled. Higher Ct values in sample pooling methods might be due to the inadequacy of samples when they are pooled and lower Ct values in pool testing might be due to the carrier effect due to the higher RNA content in the pool. There are not many studies that have commented on % CoV variability pertaining to COVID-19 testing [18].

When a person shows a weak positive result, it means that the viral load is low, In these cases we need to amplify samples several more times to detect the virus, leading to high Ct value. This kind of weak positive may be a of result of sample inadequacy or due to collection from a person in very early or late stages of infection when the viral load is low. These samples cause missed detection when they are pooled. Such weak positive samples may pose a significant threat of spreading the infection to others, particularly if undetected. Studies on the clinical significance of low viral load positive cases are needed and it remains a question of whether it is safe for larger public health efforts to miss detection of such cases.

Pooling samples can be very challenging logistically. Usually in pooled testing, if a pooled sample tests negative, then all samples within that pool are deemed negative and if a pool tests positive, then individual samples making up that pool are tested again as individual samples to identify the positive samples. Hence the larger the pool the more challenging is the deconvolution [19]. Further there is additional time required in deconvoluting larger pools, which leads to time delay in reporting, and this becomes crucial in severely ill patients and the high-risk contacts where early reporting is requisite [20]. Not to neglect the anxiety caused by isolation in patients included in the positive sample pools, until retesting of the individual samples is completed.

A lot of factors affect the sensitivity of RTq-PCR, like the sensitivity of the kit, dilution used, the techniques of sample collection, type of samples (NPS, oropharyngeal, nasal, etc.), sample transport temperature, and viral load in the sample that varies according to the stage of infection [21, 22]. Therefore the variability RT- qPCR in sample pooling strategy has its own limitations, as it cannot ensure the diagnostic integrity of the individual sample and can mask the technical errors like insufficient sampling [18, 20, 23]. This can lead to a higher percentage of false negatives, reduces sensitivity, and increased risk of missing weak viral load or borderline positive samples due to sample dilution [10, 16, 20, 24]. Although pooling of samples facilitates rational use of resources, it might miss individuals who might be positive for COVID-19 [25, 26]. This balance is important when determining the relative benefits and costs of decreased accuracy and countries and public health sectors deciding on action need to keep all these factors in mind when deciding optimal strategies.

This information becomes crucial for deciding the pooled strategy testing for disease like COVID-19. In the current pandemic, we simply cannot afford to miss any positive cases, although we also have a high demand for resources and testing that not everywhere can meet. With the highly contagious nature of SARS-CoV-2, which is represented by a high Ro value of around 3 for SARS-COV-2, every missed positive case can lead to rapid spread of infection to others and can lead to outbreaks [27].

### Limitations

Our study was conducted in a single laboratory in a defined geographical area, and we have evaluated only two different pool sizes. More studies and data are needed to validate sample pooling strategies and the clinical significance and threat of communicability of weak positive samples.

## Conclusion

Based on the study results, we conclude that pooled testing strategies miss low viral load/ with weak positive COVID–19 cases and that there is high variability in results when the samples are pooled. While in a low resource setting, where pooled sample testing is better than not having any kind of testing, sample pooling might be an effective way for mass screening and the increase in percentage of false negative tests may be dismissed. That said, caution is advised, and it needs scrutiny. In the current scenario of the pandemic, validation studies on the cost-effective analysis of pooling samples should be done, considering the cost and risk of missing even a single positive person. As COVID-19 numbers are still high and testing capacity needs to be high, we must meticulously evaluate the testing strategy of each country depending on its testing capacity, infrastructure, economic strength, and need to find optimal and effective testing strategies to limit the spread of disease.

## Data Availability

The data is available with the corresponding author Dr. Sally, Director of Biogenix G42 Lab and will be produced on request.
